# Platelet Rich Plasma for Treatment of Rheumatoid Arthritis: Case Series and Review of Literature

**DOI:** 10.1155/2020/8761485

**Published:** 2020-01-31

**Authors:** Humeira Badsha, Ghita Harifi, William D. Murrell

**Affiliations:** ^1^Dr. Humeira Badsha Medical Center, Dubai, UAE; ^2^Emirates Integra Medical & Surgery Centre, Dubai, UAE; ^3^Emirates Healthcare, Dubai, UAE; ^4^Seventh- Medical Support Unit-Europe, Kaiserslautern, Germany; ^5^Lansthul Regional Medical Center, Division Surgery, Dept. Orthopaedic Surgery, Landsthul, Germany

## Abstract

Platelet-rich plasma (PRP) is an autologous blood product with platelets above circulating levels and releases several growth factors after activation. PRP may help to decrease joint inflammation by modulating synovial cell proliferation and differentiation and inhibition of catabolic pathways in various articular conditions. Though PRP has shown good efficacy in osteoarthritis and other musculoskeletal conditions such as synovitis, epicondylitis, skeletal muscle injuries, and tendinopathy, there is limited experience for the use of PRP in patients with rheumatoid arthritis. Precise mechanisms of action of PRP are not known. We present clinical experience for treatment with PRP (2–4 ml) in four patients with rheumatoid arthritis who had inadequate response and persistent pain and inflammation with intra-articular steroids. Irrespective of past and ongoing treatments and duration of disease, all patients showed improvement in the visual analog scale and disease activity score of 28 joints at 4 and 8 weeks after injection. There was an improvement in joint inflammation on ultrasound imaging in some patients. These effects were sustained for up to 1 year. No adverse effects were reported in any patient. PRP may be a safe and useful therapy in patients with rheumatoid arthritis who fail to respond to one or more established treatment options.

## 1. Introduction

Rheumatoid arthritis (RA) is a chronic inflammatory joint disease that involves damage to the cartilage. RA shares features such as cartilage matrix degradation and progressive joint remodeling with osteoarthritis (OA), while OA joints exhibit predominant inflammation. This suggests a shared underlying pathology in RA and OA [1]. Several cytokines, chemokines, proteases, cell adhesion molecules, and angiogenic factors are common in the pathological processes in RA and OA [2]. Macrophages and macrophage-derived growth factors such as vascular endothelial growth factor (VEGF) are increased in the inflamed synovium of both RA and OA joints [2, 3]. Active angiogenesis is evident in the synovium of affected joints in both RA and OA [3, 4], and the redistribution of blood vessels in the synovial tissues may compromise cartilage metabolism and exacerbate chondropathy. RA may often coexist with OA.

Response to therapies differs in RA and OA. Though nonsteroidal anti-inflammatory drugs (NSAIDs), analgesics, and steroids are used in both conditions, biological agents such as antitumor necrosis factor (anti-TNF) therapies more convincingly reduce inflammation and angiogenesis in RA than in OA [2]. Biologic disease-modifying antirheumatic drugs (bDMARDs) that target key immunological components of disease pathology have transformed the management of RA. However, clinical and immunological response to bDMARDs is variable and inconsistent [5]. There remains an ongoing quest for therapies that target articular inflammation and also inhibit synovial angiogenesis and prevent damage to healthy cartilage.

Platelet-rich plasma (PRP) is an autologous blood sample that has highly concentrated platelets and several cell-growth factors. PRP may help to restore cartilage morphology and microarchitecture due to its action on synovial cell proliferation and differentiation and inhibition of inflammatory factors in joints [6–8]. Though PRP has shown good efficacy in OA and other musculoskeletal conditions such as epicondylitis and tendinopathy [9–12], there is limited experience for the use of PRP in patients with RA.

We present clinical experience for treatment of RA with PRP in patients who had inadequate response and persistent pain and inflammation with intra-articular steroids. We administered activated leukocyte poor PRP with two times baseline concentration of platelets in volumes of 2–4 ml. The PRP kit used was Prizhma by AK Pharma, Miami Florida, USA. The kit is a completely closed system with collection and activation tubes. The collection tube has a gel separator which facilitates removal of 100% red blood cells and more than 99% of white blood cells. The kit has an inbuilt activation mechanism through calcium chloride.

As per the MARSPILL classification, our PRP was described as M, A, RBC-P, one spin (Sp1), PL4-6, not guided G, poor (LcP), and not activated (A-MARSPILL) [13]. It was administered to four patients of RA with persistent pain and refractory inflammation in joints by ultrasound-guided (USG) injection [14]. USG has been performed for all patients. We used the “LOGIQ e” portable machine with the 12L-RS probe with a linear array for USG (General Electric Healthcare, US). All USG examinations were performed by the same operator. The scores of Visual Analog pain Scale (VAS), clinical examination, and Disease Activity Score using 28 joints (DAS 28) were recorded on the day of PRP and 1 month later. Written consent was obtained for all patients to record and report their ultrasound reports and other clinical details.

### 1.1. Case 1

A 40-year-old European female patient with a history of RA for last 5 years, presented for the follow-up visit. The patient had the antibodies-to-rheumatoid factor level of 55.23 IU/mL and cyclic citrullinated peptide of 476.4 U/mL. She had failed therapy with methotrexate (MTX) but was stable on tofacitinib for last two years. Though DAS 28 scores were suggestive of low disease activity, there was persistent inflammatory arthritis of the right wrist. The patient had received intra-articular steroid injections, but the synovitis continued to persist.

**Figure 1:**
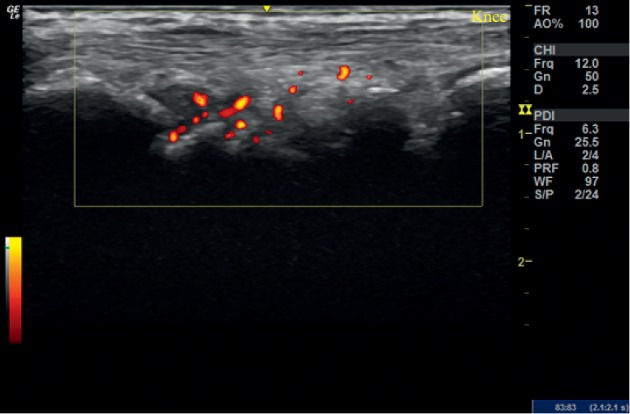
Initial ultrasound examination of the right wrist in case 1. Longitudinal view showing a power Doppler grade 2 synovitis. Multiple small carpal erosions were observed.

On her initial visit, the VAS score was 45 mm and DAS 28 score was 3.45. X-ray was not performed. USG of the right wrist showed grade 2 synovitis on Grey Scale US (GSUS) and grade 2 Power Doppler (PDUS). Multiple small carpal erosions were noted (Figure 1).

**Figure 2:**
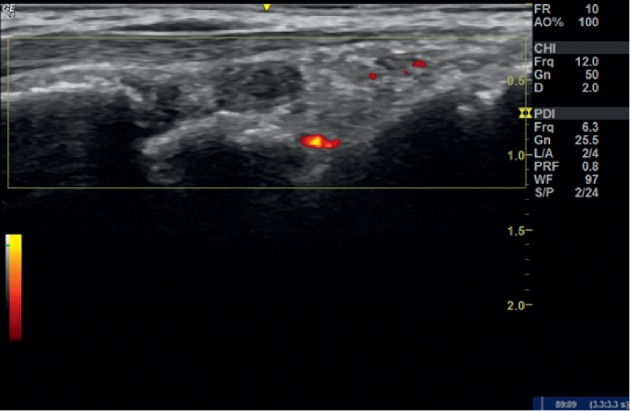
Ultrasound examination of the right wrist at 6 weeks in case 1. Longitudinal view of showing a power Doppler grade 1 synovitis.

The patient was administered with 2 ml of PRP and evaluated after 6 weeks. Her VAS score had dropped to 15 mm and DAS 28 to 1.45. There was no swelling or tenderness over the right wrist. USG showed grade 2 synovitis in the combined grading system with grade 2 synovitis on GSUS and grade 1 PDUS (50% drop in PD signal compared with the initial visit) (Figure 2). At 12 weeks, her DAS 28 was still 1.45, and USG remained the same as that on her visit after 6 weeks.

### 1.2. Case 2

**Figure 3:**
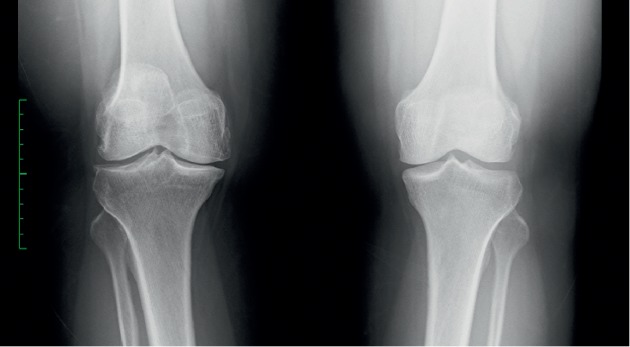
X-ray examination in case 2.

A 40-year-old Asian female patient with the antibodies-to-RF level of 40 IU/mL and CCP of 128 U/mL presented with unremitting pain and swelling in both knees. X-ray showed osteoarthritis of both knees (Figure 3). In the past, the patient was treated with MTX, azathioprine, tociliziumab, and adalimumab and currently was on golimumab and MTX. Pain VAS and DAS 28 scores of both knees were 80 mm and 4.58, respectively. USG examination (same) was suggestive of OA in both knees and no active synovitis on power doppler. PRP 4 ml was administered in both knees. At 12 weeks postinjection, her pain was relieved (VAS score of 0), and DAS 28 was 1.45. USG showed OA changes with no significant synovial hypertrophy and no power Doppler.

### 1.3. Case 3

**Figure 4:**
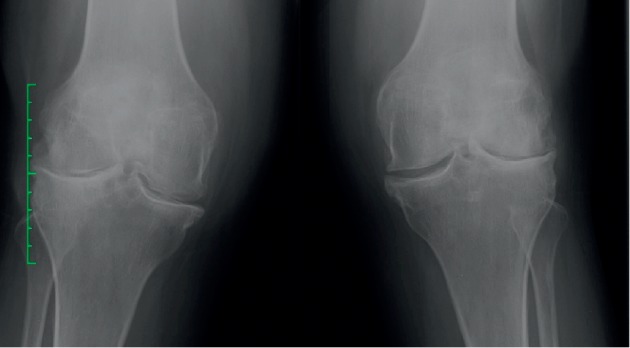
X-ray examination in case 3.

**Figure 5:**
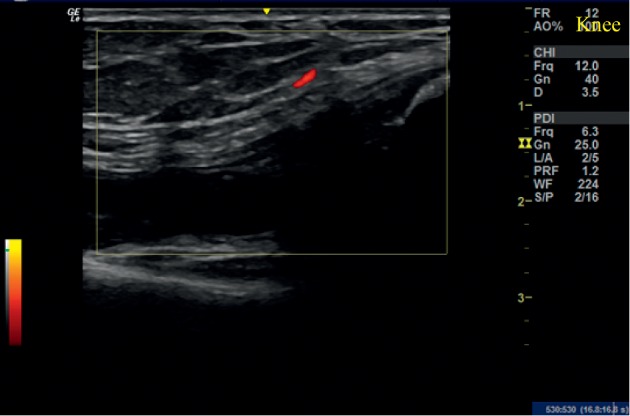
Ultrasound examination of the right knee in case 3.Longitudinal view showing initially synovitis with significant synovial effusion.

**Figure 6:**
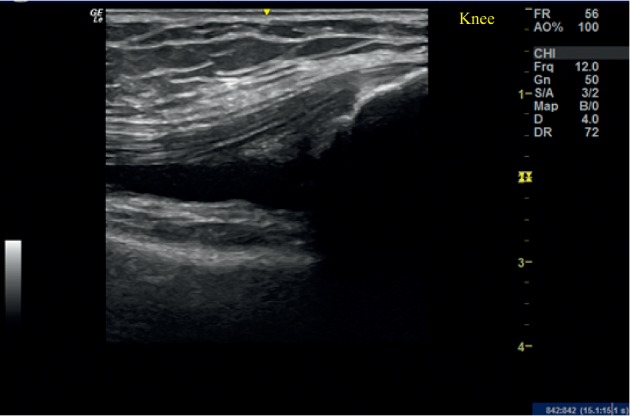
Ultrasound examination of the right knee in case 3 at 8 weeks. Longitudinal view showing synovial effusion 8 weeks after PRP injection.

A 35-year-old Asian female patient who had a history of RA for last 10 years presented with persistent pain and swelling in both knees. The patient was tested negative for RF (6.0 IU/mL), CCP (0.50 U/mL), and HLA B27. She had failed therapy with MTX, azathioprine, sulfasalazine, and rituximab and was currently on adalimumab and MTX. Her VAS score was 40 mm and DAS 28 score was 1.89 for both knees. Plain radiographs (Figure 4) demonstrated Kellgren–Lawrence (K–L) grade 4 OA changes in both knees. USG showed significant synovial hypertrophy (grade 2) exhibiting a mild PDUS signal (Figure 5). There was significant synovial effusion in the suprapatellar recess and the medial and lateral parapatellar recess. There were few small erosions on the tibial insertion of the patellar tendon. The patient was administered 4 ml PRP into each knee. At 8 weeks of follow-up, her VAS score remained 40 mm, and DAS 28 score was 1.85. On USG, significant synovial effusion persisted. However, synovial hypertrophy was slightly reduced from grade 2 to grade 1 (Figure 6).

### 1.4. Case 4

**Figure 7:**
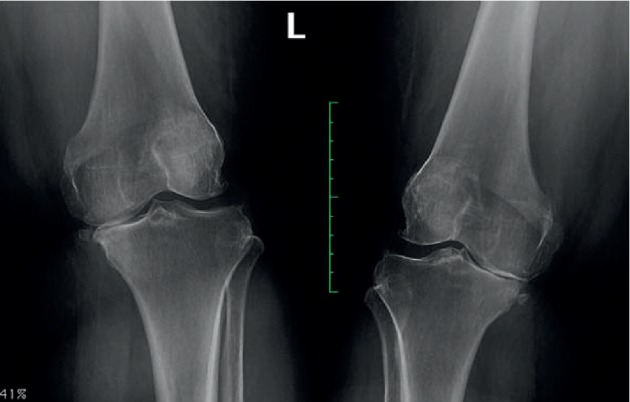
X-ray examination in case 4.

A 54-year-old Asian lady who had RA for the last 15 years had stage K–L grade 4 OA on X-rays of both knees (Figure 7). The patient had antibodies-to-rheumatoid factor level of <20 IU/mL and cyclic citrullinated peptide of <5 U/mL. USG showed grade 2 synovial hypertrophy and synovial effusion and negative PD. She was on MTX and prednisolone. Her VAS score was 80 mm, and DAS 28 score was 3.78 for both knees. She received PRP injections 4 ml in both knees. After 4 weeks, her VAS had decreased to 50 mm and 70 mm in the right and left knees, respectively. She was given repeat injections of PRP in both knees. At 4 weeks following the second dose, VAS scores were 50 mm in both knees, and DAS 28 score improved to 2.96. She was followed up at 1 year after the injection, and her VAS and DAS scores were sustained at the same levels during this period. USG showed improvement on synovial hypertrophy to grade 1 and decrease in effusion both at 1 month and 1 year following the second dose of PRP.

## 2. Discussion

RA is the most common immune-mediated chronic inflammatory joint disorder which affects around 1% of the population [15, 16]. Articular inflammation and synovial hyperplasia are the key pathological changes in RA. There is activation of the fibroblast-like synoviocytes (FLS), and the growing synovium invades into the adjacent bone and cartilage. The FLS produce various cytokines, chemokines, and matrix-degrading enzymes that lead to progressive articular degradation [17].

Treatment of RA with steroids, DMARDs, biologic agents, tofacitinib, and lifestyle modifications helps to reduce pain and inflammation and improve Quality of Life for patients [15]. However, patients may fail treatment with one or more of these agents. RA shares features such as articular erosion and inflammation with OA, a condition where benefits of PRP are reported [10, 18]. The number of platelets correlates with rheumatoid activity, and PRP regulates migration, invasion, and adhesion of FLSs on the extracellular matrix by influencing the reorganization of actin cytoskeleton and increasing the expression of matrix metalloproteinase-1 [19]. PRP is a volume of plasma fraction of autologous blood with platelet concentrations above baseline [20]. There is no uniform concentration of platelets established for PRP; concentrations are reported to range from 3,00,000 to 1,000,000 platelets/*μ*L [18, 21]. PRP has diverse clinical applications in wound healing and tissue regeneration. This can be attributed to various growth factors and cytokines in PRP, for example, platelet-derived growth factor, epidermal growth factor (EGF), connective tissue growth factor (CTGF), platelet factor-4, vascular endothelial growth factor (VEGF), transforming growth factor-*β*, insulin-like growth factor-1, interleukin 1*β*, and IL-6 [20, 22–24].

Benefits of PRP for repair and regeneration of articular cartilage have been confirmed in preclinical studies. In cell cultures of chondrocytes, PRP increases cellular and cartilage matrix proliferation and synthesis of proteoglycans and collagen type II as well as downregulates catabolic mediators such as IL-1*β*, TNF-*α*, IL-6, and MMP-13 directly and indirectly [25–27]. Clinical benefits of PRP have been reported for several musculoskeletal conditions including tendinopathies, bone defects, and other articular pathologies [18, 28]. PRP has been evaluated for symptomatic knee OA [10, 29]. PRP has demonstrated beneficial effects in OA by facilitating tendon and articular bone repair and elimination of inflammation. In 24 elderly patients, monthly intra-articular PRP injections decreased pain and volume of synovial fluid and favorably impacted the component proteins in the synovial fluid in elderly patients with mild-to-moderate knee OA and suprapatellar bursitis [30]. After 2 injections, there was a significant decrease in Lequesne index values. There was a decrease in proinflammatory proteins such as apolipoprotein A-I, haptoglobin, matrix metalloproteinase, immunoglobulin kappa chain, and transferrin and an increase in proteins such as matrilin, transthyretin, and complement 5 which help combat the degenerative processes.

PRP has demonstrated comparable benefits to hyaluronic acid for the treatment of mild-to-moderate knee OA. In a randomized controlled trial, patients receiving PRP (*n* = 49) and hyaluronic acid (*n* = 50) reported no significant differences in the Western Ontario and McMaster Universities Osteoarthritis Index (WOMAC) pain scores when followed up over a period of one year. However, mean VAS scores were significantly lower in the PRP group at both 24 (34.6 ± 3.24 vs. 48.6 ± 3.7; *P*=0.0096) and 52 (44 ± 4.6 vs 57.3 ± 3.8; *P*=0.0039) weeks. At 12 weeks, the PRP group had a greater decrease in proinflammatory cytokines interleukin 1*β* (0.14 ± 0.05 pg/mL vs. 0.34 ± 0.16 pg/mL; *P*=0.06) and TNF-*α*(0.08 ± 0.01 pg/mL vs. 0.2 ± 0.18 pg/mL; *P*=0.068) [9].

PRP is a safe and useful therapy in patients with haemophilia who develop chronic synovitis. In the treatment of 2 ankle, 7 elbow, and 19 knee joints, PRP (mean volume of 4 ml) significantly reduced the Haemophilia Joint Health Score (HJHS) and numbers of bleeding episodes in 19 patients of haemophilia. All patients reported relief in pain and improvement in VAS scores [31].

We report the use of PRP in RA patients who had failed therapy with one or more treatment options for RA. In our experience, intra-articular PRP (4 ml) resulted in improvement in VAS and DAS 28 scores at 4 and 8 weeks after injection. The effects were sustained for up to 1 year of follow-up. These clinical effects of PRP were independent of the duration of disease and past or ongoing treatment for RA. In addition, all patients showed a decrease in synovial hypertrophy and effusion on USG examination. We observed variable responses to PRP injections in four patients with RA. Of the three patients who improved clinically, only 2 had synovitis on USG, and this is consistent with anecdotal communications of results of the application of PRP in RA. The clinical benefits of PRP in RA can potentially be explained by anti-inflammatory effects. PRP suppresses catabolic pathways and results in increase in chondrogenic markers in various preclinical cellular preparations. In human nucleus pulposus cell lines, PRP countered the lipopolysaccharide-induced inflammatory markers (IL-1*β*, TNF-*α*, and MMP-13) and restored the chondrogenic proteins such as collagen II, phosphorylated SOX9 (p-SOX9), and CD44 [32]. PRP treatment has also improved disc height, reduced histological degeneration, and increased MRI signal intensity in animal experiments for low back pain [33, 34]. Pain relief with PRP is reported in patients with skeletal muscle injuries and strains [35–37]. There is also evidence for reduction of inflammation and size of fibrotic scars with PRP in skeletal muscle injury [38].

No adverse reactions were noted over the mean 5-month follow-up period (2–12 months) in the four patients who received intra-articular treatment with PRP. Similar safety of PRP has been reported in discogenic low back pain [39] and degenerative meniscal tear in knee [40]. There are no serious adverse events reported for PRP in literature [41]. The safety of PRP can be explained by the autologous origin of the platelets.

Theoretically, there is no risk for immunogenic reactions, and no systemic effects have been reported [42]. Bovine thrombin, when used for activation, may lead to hypersensitivity reactions. However, modern preparations are not produced by using bovine thrombin. However, safety of PRP has not been evaluated in well-planned clinical studies for musculoskeletal conditions [24].

The precise mechanism of action of PRP is not clearly known. Autologous plasma rich in platelets is believed to be a rich source of growth factors from the harvested platelets that have been activated by endogenous thrombin due to the added calcium chloride in the PRP preparations [43]. Platelet activation results in growth-factor release [44]. These growth factors accelerate healing and improve functional outcomes in articular injuries. Several growth factors component to PRP have a role in angiogenesis. These include EGF, VEGF, CTGF, and keratinocyte growth factor. This explains the acceleration of healing with PRP in articular conditions. In addition, EGF regulates secretion of collagenase, and CTGF has a role in cartilage regeneration. Together, these growth factors can help to repair articular structure and restore joint function [24].

Our findings suggest a beneficial role of PRP in patients with RA, in particular patients who fail therapy with one or more of other agents. However, these findings should be confirmed in well-planned clinical studies for RA. Further studies should be conducted to understand the impact of PRP volume, platelet concentration, severity of RA, and other comorbid conditions on the use of PRP in patients with RA.

We have administered activated leukocyte poor PRP with approximately two times of baseline concentration of platelets in volumes of 2–4 ml to 4 patients in our clinical settings. There is a growing need for the standardization of preparation and application of PRP [6, 45]. Cellular composition and protein constituents may differ in various PRP preparations. Based on the method of preparation of PRP, the American Association of Blood Banks has defined PRP as the plasma fraction resultant of a single light spin of whole blood which is richer in platelets in comparison with other cell types. Red blood cells and leukocytes are greatly reduced in PRP preparations [46]. Double-spin methods are also reported for PRP preparations [47]. Growing experience with PRP therapeutics necessitates use of more standard and homogenous preparations to maximize clinical benefits.

## 3. Conclusions

PRP may be a safe and useful therapy in patients with RA who fail to respond to established treatment options with progression of disease. Intra-articular PRP may be easier to adopt in patients with RA as this is administered in outpatient settings and does not require hospitalization. PRP may evolve as a surgery-sparing option in patients with RA. However, PRP preparations need to be handled with caution to avoid any inadvertent transmission of infections. PRP should be evaluated in clinical trials for improvement in joint structure and composition and restoration of function and Quality of Life in patients with RA. Treatment with NSAIDs and steroids have efficacy for relief in joint pain and stiffness, but these do not influence disease progression. DMARDs gained importance for attenuation of disease progression though long-term remissions have not been adequately achieved in patients with RA. Long-term, well-planned studies are warranted to confirm if PRP preparations can check progressive disability in RA.
